# A Learning Probabilistic Boolean Network Model of a Manufacturing Process with Applications in System Asset Maintenance

**DOI:** 10.3390/e27050463

**Published:** 2025-04-25

**Authors:** Pedro Juan Rivera Torres, Chen Chen, Sara Rodríguez González, Orestes Llanes Santiago

**Affiliations:** 1 Departmento de Informática y Automática, Universidad de Salamanca, Patio de las Escuelas 1, 37006 Salamanca, Spain; srg@usal.es; 2 Escuela Técnica Superior de Ingeniería Industrial de Barcelona, Universidad Politécnica de Cataluña, Av. Diagonal, 647, 08028 Barcelona, Spain; 3 St. Edmund’s College, University of Cambridge, Mount Pleasant, Cambridge CB3 0BN, UK; 4 Department of Computer Science and Technology, University of Cambridge, Cambridge CB3 0FD, UK; 5 Departamento de Automática y Computación, Universidad Tecnológica de La Habana José Antonio Echeverría (CUJAE), Marianao, La Habana 11500, Cuba

**Keywords:** fault detection and isolation, machine learning algorithms, probabilistic Boolean networks, probabilistic Boolean network modeling

## Abstract

Probabilistic Boolean Networks (PBN) can model the dynamics of complex biological systems, as well as other non-biological systems like manufacturing systems and smart grids. In this proof-of-concept paper, we propose a PBN architecture with a learning process that significantly enhances fault and failure prediction in manufacturing systems. This concept was tested using a PBN model of an ultrasound welding process and its machines. Through various experiments, the model successfully learned to maintain a normal operating state. Leveraging the complex properties of PBNs, we utilize them as an adaptive learning tool with positive feedback, demonstrating that these networks may have broader applications than previously recognized. This multi-layered PBN architecture offers substantial improvements in fault detection performance within a positive feedback network structure that shows greater noise tolerance than other methods.

## 1. Introduction

Probabilistic Boolean Networks (PBNs) [1,2] have been applied in the scientific literature not only to model Gene Regulatory Networks (GRNs) but also in engineered systems, such as smart power grids and manufacturing systems. PBNs are stochastic adaptations of Kauffman’s Boolean Network (BN) model [3,4], where a GRN is represented as a graph with each gene depicted as a binary node that can either be in an “expressed” or “unexpressed” state. Each node’s value is determined by a Boolean function (a predictor) based on its current state. Unlike BNs, PBNs are not deterministic. Each node in the network has one or more predictors, each associated with a probability, effectively creating a probabilistic tree of BNs selected by likelihood. BNs and PBNs tend to evolve towards certain states, either a single state (fixed point) or a set of cyclic states, known as attractors, which represent the network’s behavior in a steady state. These attractors can be seen as “knowledge” stored within the network. Past research has shown that these states in a manufacturing system are analogous to machine states, in normal operation or in various fault or failure conditions, making these states equivalent to intervention strategies like predictive or preventive maintenance [5,6,7,8,9,10]. Machine learning has been used to control PBN models and guide their “learning” through Deep Reinforcement Learning [11]. The capability of PBNs to engage in basic Reinforcement Learning has been previously explored in [5]. In this paper, building on the learning processes inherent to neural networks, we propose an alternative PBN architecture designed to preserve a state without external intervention—the paper’s first contribution. The proposed architecture is parameterized, enabling fine-tuning to optimize network performance, which forms the second contribution. The results demonstrate the method’s effectiveness and present new possibilities, as no previous method of this kind has been reported. This work is a continuation of work we have been developing in [5,12,13,14].

## 2. Materials and Methods

### 2.1. Probabilistic Boolean Networks

**Figure 1:**
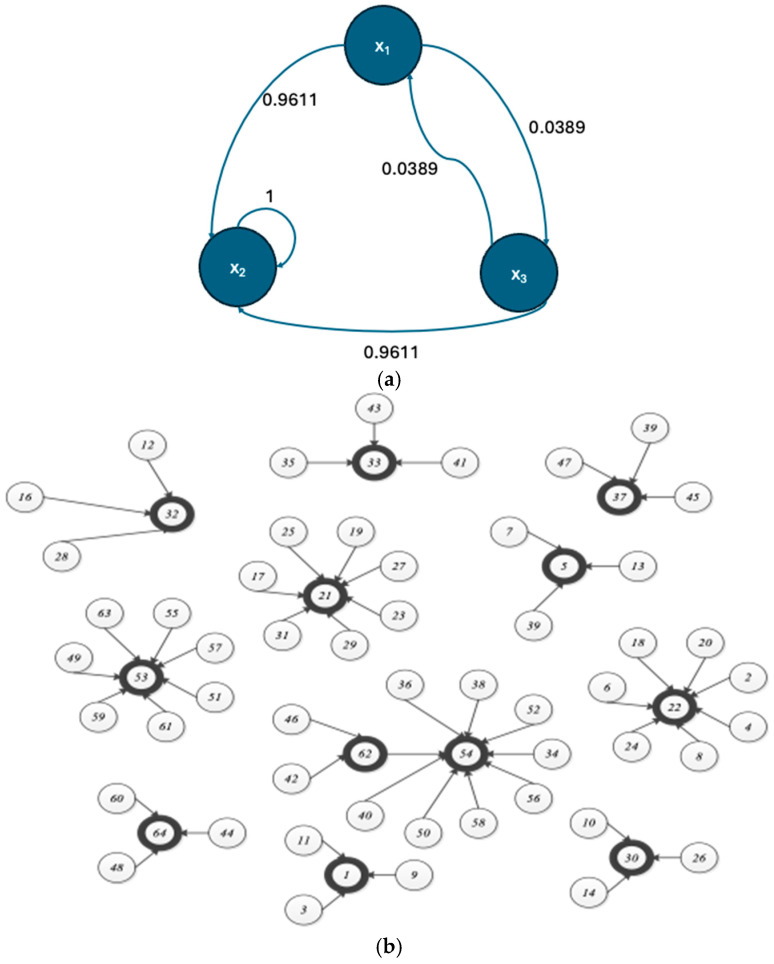
(**a**) A Probabilistic Boolean Network; (**b**) a transition diagram of one of the constituent BNs of a Probabilistic Boolean Network.

Probabilistic Boolean Networks (PBNs) are models of state-transition dynamical systems widely employed in the modeling of Gene Regulatory Networks. They represent a non-deterministic extension of Kauffman’s Boolean Network (BN) model and were introduced by Shmulevich and Dougherty [1,4]. Figure 1a illustrates an example of a PBN, while Figure 1b shows one of its constituent Boolean Networks, in which the dark circles denote attractors (i.e., recurring states).

PBNs serve as computational frameworks for representing and studying the dynamics of complex systems. These models operate in discrete time and are defined by a set of Boolean variables alongside their probabilistic transition behavior.

Let $V=\left\{x_{1},x_{2},...,x_{N}\right\}$ denote a collection of Boolean variables, where each $x_{i}$ can assume binary values 0 or 1 (OFF or ON). A PBN is formally defined as a graph *G*(*V*,*F*), where *V* is the set of nodes and *F* is a collection of predictor function sets *F* = ($F_{1},...,F_{n\;})$. Each $F_{i}=\left\{f_{1}^{\left(i\right)},\;f_{2,}^{\left(i\right)},\;...,\;f_{l(i)}^{\left(i\right)}\right\}$ comprises l(i) predictor functions, with each $f_{l(i)}^{\left(i\right)}:\;{\left\{0,\;1\right\}}^{v}\to \left\{0,\;1\right\}$, where *v* denotes the number of inputs into the node *x_i_*.

At any moment, a context within a PBN is determined by selecting a specific set of predictor functions. With *D* possible contexts, there are *D* vector functions $f_{1},...,f_{N}$, where each $f_{j}=(f_{1}^{\left(i\right)},...,f_{l(i)}^{\left(i\right)})$, where $1\leq j\leq N\;\;and\;1\leq j_{i}\leq l(i),\;f_{j_{i}}^{\left(i\right)}\in F_{i}$. A context in a PBN maps the current Boolean vector of node states to a new one using $f_{j}:\{0,1\}\;\to \{0,1\}$, and each context corresponds to one of the constituent Boolean Networks that governs the system at a given time.

There is a probability associated with the selection of a particular predictor function for each node, and also with the choice of an overall context or constituent BN. Specifically, $c_{j}^{\left(i\right)}=Pr\{F^{\left(i\right)}=f_{j}^{\left(i\right)}\}$, represents the selection probability. If $F_{i}$ defines a specific context, the total number of contexts in a PBN (i.e., the number of constituent Boolean Networks) equals the product of the number of predictor functions across all nodes. For instance, in a two-node PBN with two predictors per node, there would be *D* = 4 total contexts. The probability of choosing any given context is then the product of the individual selection probabilities for that context. For a full and formal definition of a Probabilistic Boolean Network, see references [1,2,3,4].

### 2.2. Machine Learning

Machine learning (ML) is a branch of Artificial Intelligence (AI) that focuses on developing algorithms that learn and improve from the data they process. ML algorithms analyze data, identify patterns, and use this knowledge to enhance task performance, with varying levels of human intervention. The main types of ML strategies are supervised learning, unsupervised learning, semi-supervised learning, and reinforcement learning. In supervised learning, the system learns from a labeled dataset, mimicking human learning. In unsupervised learning, the system works with unlabeled data, aiming to identify patterns through clustering, density estimation, anomaly detection, or dimensionality reduction. Semi-supervised learning uses a partially labeled dataset, while reinforcement learning involves an agent observing its environment and adjusting actions based on feedback. This study focuses on using positive (feed-forward) feedback to guide the learning process, like PBN models. Artificial Neural Networks (ANNs), a key ML method, are inspired by biological neural networks. ANNs consist of interconnected layers, where the first layer receives input, and subsequent layers perform computations to generate predictions. Neurons in these networks process weighted inputs, apply an activation function, and pass the output to other neurons, a process called forward propagation. ANNs are trained by adjusting weights and biases to minimize prediction errors using techniques like backpropagation and gradient descent. This iterative process allows the network to learn and improve its predictions over time.

### 2.3. Description of the Method

**Figure 2:**
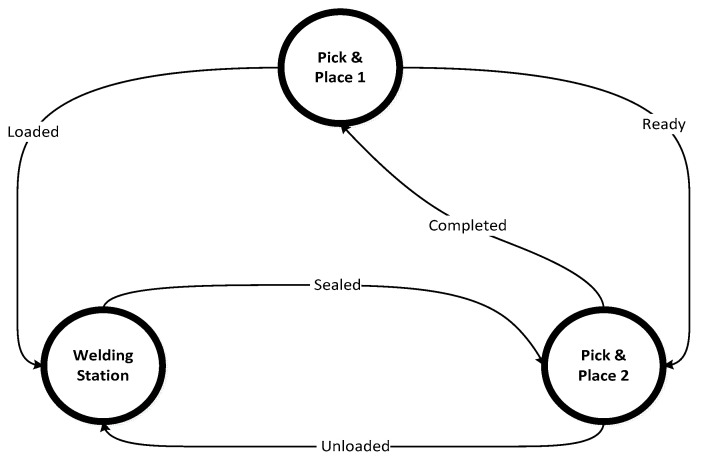
Ultrasonic welding process from [11].

We use a PBN-based model established for fault detection and isolation (FDI) in a manufacturing system [6] with an Ultrasound Welding Station (WS) and two Pick and Place (PP) machines. The specific details of this model are presented in [6,7,8,9,10]. The WS joins parts, while the PP machines load and unload parts. The FDI method uses separate models for normal operation and faults, with PBNs self-organizing into attractor states related to failure modes. The failure modes of the system have been described in [6,7]. Figure 2 illustrates the process.

The method proposed here adapts the FDI approach from [15] using separate models for normal operation and each type of fault. PBNs self-organize into attractor states that correspond to different system failure modes. This model was built into the PRISM Model Checker [16], using its programming language. The advantage of using PRISM is that we can perform experiments and simulate events in a single environment. PRISM has been used for programming and analyzing the formal correctness of several PBN models [5,6,7,8,9,10]. For the selection probabilities of the predictors of the model, an unbiased approach has been used as a starting point (note that this is unrelated to the concept of bias in ANNs), where the probabilities are all balanced, each predictor receiving an equal probability with respect to the other (the node has a 0.5 probability of producing a ‘0’ or ‘1’). The values of the probabilities for each predictor are set at the start of the execution, when the input values for the first level are chosen (per the patterns previously mentioned) by the user. The output values of each layer are based on the input values selected, and the values resulting from the application of the predictor functions. The values of the output layer’s input correspond to the output of the inner layer just before. At this point, the value of the output of each node in the output layer is compared to the value from the input pattern that the user would like the network to learn (the value for the node that the user wants). If that value is correct, the probability of the selected predictor for that node is strengthened (increased), and the value of the predictor(s) that produce an incorrect result are lessened by a small factor delta that is constant and set at the beginning of the execution. For the internal layers, there are no values that can be used to compare each layer’s output, and an immediate assessment of the correctness of the output of each internal layer cannot be performed. For this reason, a correction factor is introduced at the output of all layers. We cannot compare these values, but we perform a computation to measure each of the output node value’s correctness. The correction factor is a linear function that has a value of 1 if all node values are correct, −1 if all node values are incorrect, and 0 if the number of correct values is equal to the number of wrong values. The delta factor is then multiplied by this correction factor, which is added to the probability of the output of every node. The proposed architecture is a PBN, as defined by Shmulevich and Dougherty in [1], where each node represents a machine in the system. This architecture features a multi-layered feed-forward structure, with each layer consisting of a PBN. Each node has predictor functions that determine its next state based on the influence of the network’s previous state. All connections are fixed, reflecting the physical structure of each network, with connections intentionally aligned to their specific connection patterns. This structural modification, implemented in PRISM, represents a feed-forward PBN, distinguishing it from the model in [6]. The network consists of four layers: an input layer, two middle layers, and an output layer. To assess the system’s performance, a Performance Index (PI) is defined as

(1) $$PI(t)=\frac{\sum_{\tau =t-n+1}^{t}nocb(\tau )}{n}$$

where *nocb* represents the number of correct bits after each iteration *τ*, and $n\leq \;Min\{\bar{n},\bar{\tau }\}$, with $\bar{\tau }$ being the number of iterations with the same input pattern and $\bar{n}$ being a user-defined constant. This index tracks the algorithm’s learning progress, providing the average number of correct bits in the last *n* iterations, where *n* is the smallest value between $\bar{n}\;$and the number of iterations with the same input pattern. Two values of $\bar{n}$ were tested: $\bar{n}$ = 100 and $\bar{n}$ = 200. A Satisfaction Index (SI) was introduced, where an input pattern remains fixed until the PI meets the desired SI. The complete input set is sought by cycling through all possible input combinations. Once the set is complete, the SI is raised to a higher satisfaction threshold, typically between 0.55 and 0.85. The number of iterations needed to complete the input set varies. Testing the equality function simulates intervention post-fault detection, akin to predictive maintenance. In these networks, positive feedback replaces backpropagation, and transition probabilities are adjusted based on the PI, SI, and a correction factor to select the appropriate BN for learning. The delta parameter, set initially at 0.01, is introduced as a correction factor to modulate the probability of predictor occurrences. The inner and output layers’ correction factors are determined as described earlier. Simulations were carried out using PRISM, following the method outlined but configured as a positive feed-forward PBN with an input layer, two middle layers, and an output layer. The algorithm tracks the steps performed, calculates iterations to achieve a given satisfaction level, and determines the number of complete input sets. In a biased PBN, one predictor is favored with a higher probability (depends on delta), whereas in an unbiased PBN, predictors have equal occurrence probability (0.5). The PI monitors the architecture’s performance, calculating the average number of correct input values over the last *n* iterations, where *n* is the smallest constant between $\bar{n}$ and the iterations with the same input pattern. In these experiments, the SI reflects the desired performance level. An input pattern stays fixed until the PI reaches the SI, at which point the SI is raised.

## 3. Results

We present here the results of the experiments designed to measure the performance of the proposed architecture, and a comparison between prior PBN modeling and the proposed method. The following tables present experiments that we applied to a PBN model of a larger smart grid test system, to test the performance and assess the learning capacity of the network. In Table 1 the results from experiments in PRISM evaluating the model’s learning capacity are presented. These experiments start with an initial Satisfaction Index (SI) of 0.55, which is incremented after each complete set of inputs until reaching the target SI. Here, $\bar{n}=100$ and *δ* = 0.01.

Table 1 displays the algorithm’s performance when learning the equality function with an unbiased predictor Boolean Network (PBN) where *δ* = 0.01 and $\bar{n}=100$. The SI (Satisfaction Index) values represent the final SI reached. NI stands for the number of iterations needed to achieve each target SI. NCS represents the Number of Complete Sets of inputs required to reach each SI. The predictor probability is unbiased, meaning each predictor has an equal likelihood of selection. This table shows that the initial SI of 0.55 is incrementally raised to 0.85 in steps of 0.1.

Subsequently, Table 2a,b show the impact of changing the predictor selection probabilities, introducing a bias favoring one of the two predictors by 0.01. The results indicate varying impacts on the NI and NCS across different SI ranges and biases, reflecting that biased selection probabilities can affect the transitions in learning but do not definitively impact the overall performance. Further experiments tested the variations in the $\bar{n}$ and δ parameters.

Table 3 documents the performance under $\bar{n}=200$ for an unbiased case. Overall, fewer NIs and NCS were required in most ranges, except at a final SI of 0.95, suggesting that parameter adjustments may reduce computational effort without compromising learning.

Table 4, Table 5 and Table 6 show additional performance metrics under adjusted $\bar{n}$ and *δ* values, indicating that further reductions in NI and NCS are possible. To validate these findings, a comparison with the PBN model of the system without learning was conducted, evaluating the probability of fault occurrence, implying that predictive maintenance could be feasible with an effective learning system model. This comparison underscores the potential of the Learning PBN model to maintain normal system operations and reduce fault probabilities effectively. In these experiments, the network generally required fewer iterations (NI) and complete sets (NCS) across most SI ranges, except at the final SI level of 0.95. For an initial SI of 0.65, the network with $\bar{n}=100$ required fewer NIs, while the network with $\bar{n}=200$ produced a higher NCS. Interestingly, at an initial SI of 0.75, the opposite trend occurred, with fewer NIs seen in the $\bar{n}=200\;$network, but different NCS results. Here, the final SI = 0.85 showed a higher NCS than the final SI = 0.95. Both network configurations successfully learned the function in all cases presented. Performance metrics for the unbiased case with a delta parameter of 0.001 and $\bar{n}=100$ are detailed in Table 4.

For the values in Table 5, with these parameters, the networks generally required fewer iterations (NI) and fewer complete sets (NCS) for both functions.

The results for the biased case with a delta parameter of 0.0001 and $\bar{n}=100$ are presented in Table 6. Adjusting the delta parameter to 0.0001 generally resulted in fewer iterations (NI) and complete sets (NCS) across both functions, with exceptions mainly at the final Satisfaction Index (SI) of 0.95.

**Figure 3:**
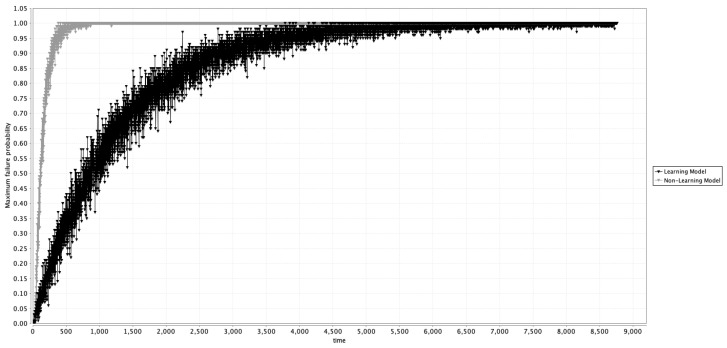
Comparison of the maximum probability of occurrence of a failure of the system using the learning model (black) and the non-learning model.

To validate our assumptions, an additional experiment was conducted using PRISM to determine the maximum probability of fault occurrence. This involved a comparison between a PBN model of the system without learning (previously used by this group in other research [5,6,7,8,9,10]) and the proposed Learning PBN model. If the Learning PBN model performs as expected, its maximum fault occurrence probability would be comparable to performing predictive maintenance (PdM) within the system, effectively reducing the likelihood of faults. This outcome aligns with the behavior observed when the proposed model successfully learns the equality function, which represents all system components operating in their normal state. Figure 3 presents the results of an experiment in PRISM to compare the maximum probability of occurrence of a failure of the two models, the Learning and the Non-Learning PBN models. From a visual inspection, the Learning PBN model reduces the failure occurrence of the system.

In these experiments, time is measured in hours on the *y*-axis, representing the maximum probability of system failure. The model simulates one year (8760 h) to show how failure probability increases without maintenance interventions. Maintenance types include reactive, preventive (calendarized), and predictive (PdM), where PdM intervenes only when needed based on the system’s condition, not on a schedule. In prior studies, maintenance was seen as a perturbation or intervention mechanism in PBNs [5,6,7,8,9,10].

**Figure 4:**
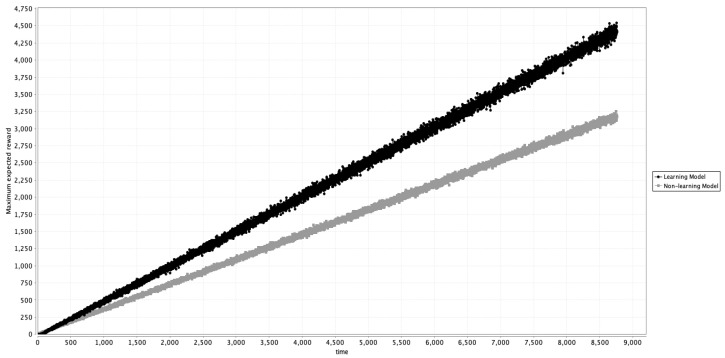
Comparison of the Maximum Expected Reward of the system using the learning model (black) and the non-learning model.

Preventive maintenance (PM) follows a schedule to slow degradation, while PdM targets intervention only when component conditions demand it, aiming to keep the system in normal operation by predicting faults. This approach minimizes unplanned failures, reduces maintenance costs, improves safety, and extends equipment life. The architecture proposed in this study optimizes the predictor functions in PBNs, allowing operators to model, predict, and proactively manage system health. PdM thus prevents failures while enabling an exploration of the effects of certain “at-risk” states within the failure basin, where some components may operate normally, while others with lower priority remain in a fault state. Figure 4 presents another comparison. This time, we have assigned a reward of ‘1’ for every time the simulation achieves the normal operation state, and our purpose here is to determine which of the two models achieves a higher expected reward. The learning model achieved a higher reward, which means it learned the normal operating state more than the non-learning model, thus achieving its purpose.

## 4. Conclusions

The novel architecture in this study advances the application of PBNs in engineering modeling by enabling autonomous learning to maintain a set of inputs, thus improving the model performance and enhancing the predictive accuracy. Unlike conventional learning techniques that adjust weights via backpropagation, PBN learning modifies predictor probabilities to influence network transitions, a process that can lead to faster learning and may even allow hardware implementation in ASICs. Negative feedback systems, in contrast, often need extensive training data and are less tolerant to noise [17].

Our initial experiments confirm that the network can learn the equality function, highlighting areas for further research, including the selection of probabilities and the impact of parameters *δ* and $\bar{n}$. Additionally, factors like the network structure, synchronicity, and attractor selection require in-depth analysis [18,19]. A major limitation of the current setup is the scalability of PRISM, which, despite its analytical strengths, lacks the capacity for large-scale processing. Exploring alternative environments with better scalability is essential to advancing this architecture.

While PBNs differ from ANNs, these findings suggest potential parallels that warrant further investigation. This research aims to deepen the understanding of learning in PBN-modeled systems, with future applications in predictive maintenance. Such models could optimize maintenance schedules, enhance resource use, and lower operational costs by refining predictor probabilities to sustain stable, learned states. This method promises improved maintenance models, driving system efficiency and reliability.

## Figures and Tables

**Table 1:** Performance of the algorithm learning the equality function using an SI (unbiased PBN, δ=0.01, $\bar{n}=100$).

Final SI	Initial Satisfaction Index (SI)							
	SI = 0.55		SI = 0.65		SI = 0.75		SI = 0.85	
	NI	NCS	NI	NCS	NI	NCS	NI	NCS
0.65	90	80	--	--	--	--	--	--
0.75	97	98	22	11	--	--	--	--
0.85	198	469	77	133	54	41	--	--
0.95	270	675	92	693	144	180	4	12

**Table 2:** (**a**) Performance of the algorithm learning the equality function using an SI for a biased PBN on component reliabilities, δ=0.01, $\bar{n}=100$, with biased predictors (first bias). (**b**) Performance of the algorithm learning the equality function using an SI for a biased PBN on component reliabilities, δ=0.01, $\bar{n}=100$, with biased predictors (second bias).

**(a)**								
**Final SI**	**Initial Satisfaction Index (SI)**							
	**SI = 0.55**		**SI = 0.65**		**SI = 0.75**		**SI = 0.85**	
	**NI**	**NCS**	**NI**	**NCS**	**NI**	**NCS**	**NI**	**NCS**
0.65	13	4	--	--	--	--	--	--
0.75	22	20	31	6	--	--	--	--
0.85	34	55	35	17	18	11	--	--
0.95	222	1233	69	278	20	88	40	30
**(b)**								
**Final SI**	**Initial Satisfaction Index (SI)**							
	**SI = 0.55**		**SI = 0.65**		**SI = 0.75**		**SI = 0.85**	
	**NI**	**NCS**	**NI**	**NCS**	**NI**	**NCS**	**NI**	**NCS**
0.65	96	64	--	--	--	--	--	--
0.75	108	101	175	78	--	--	--	--
0.85	223	611	626	516	115	35	--	--
0.95	139	276	368	511	135	76	216	260

**Table 3:** Performance of the algorithm learning the equality function using an SI for an unbiased PBN, δ=0.01, $\bar{n}=200$.

Final SI	Initial Satisfaction Index (SI)							
	SI = 0.55		SI = 0.65		SI = 0.75		SI = 0.85	
	NI	NCS	NI	NCS	NI	NCS	NI	NCS
0.65	14	10	--	--	--	--	--	--
0.75	42	77	19	4	--	--	--	--
0.85	84	266	56	86	51	23	--	--
0.95	109	756	189	705	342	517	56	90

**Table 4:** Performance of the algorithm learning the equality function using an SI for an unbiased PBN, δ=0.001, $\bar{n}=100$.

Final SI	Initial Satisfaction Index (SI)							
	SI = 0.55		SI = 0.65		SI = 0.75		SI = 0.85	
	NI	NCS	NI	NCS	NI	NCS	NI	NCS
0.65	21	2	--	--	--	--	--	--
0.75	65	119	8	1	--	--	--	--
0.85	87	263	20	23	43	27	--	--
0.95	88	479	92	332	102	50	152	67

**Table 5:** Performance of the algorithm learning the equality function using a Satisfaction Index for an unbiased PBN, δ=0.001, $\bar{n}=200$.

Final SI	Initial Satisfaction Index (SI)							
	SI = 0.55		SI = 0.65		SI = 0.75		SI = 0.85	
	NI	NCS	NI	NCS	NI	NCS	NI	NCS
0.65	8	5	--	--	--	--	--	--
0.75	26	41	12	12	--	--	--	--
0.85	33	49	57	99	22	16	--	--
0.95	131	866	62	141	28	309	427	185

**Table 6:** Performance of the algorithm learning the equality function using a Satisfaction Index.

Final SI	Initial Satisfaction Index (SI)							
	SI = 0.55		SI = 0.65		SI = 0.75		SI = 0.85	
	NI	NCS	NI	NCS	NI	NCS	NI	NCS
0.65	9	12	--	--	--	--	--	--
0.75	10	11	68	34	--	--	--	--
0.85	59	112	85	100	107	55	--	--
0.95	91	1220	472	1119	139	152	53	44

## Data Availability

The data are unavailable due to third party privacy restrictions.
